# Survival and Radiation Dose Differences Between Single Versus Multi-Session Gamma Knife Stereotactic Radiosurgery in Patients Treated for Multiple (≥10) Brain Metastases

**DOI:** 10.7759/cureus.46901

**Published:** 2023-10-12

**Authors:** Cassandra Stambaugh, Andy Y Wang, Edward Kim, John E Mignano, Christopher S Melhus, Rahul Rodrigues, Kathryn Huber, Nathaniel Stambaugh, Julian Wu

**Affiliations:** 1 Radiation Oncology, Tufts Medical Center, Boston, USA; 2 Neurosurgery, Tufts University School of Medicine, Boston, USA; 3 Mathematics, Dexter Southfield School, Brookline, USA; 4 Neurosurgery, Tufts Medical Center, Boston, USA

**Keywords:** multi-met, dosimetry, brain metasteses, radiosurgery, gamma knife

## Abstract

Objective: To explore whether treatment with multiple Gamma Knife sessions (mGK) resulted in different survival outcomes or cumulative radiation doses compared to single session Gamma Knife (sGK) in patients who have been treated for ≥10 brain metastases (BMs).

Methods: Thirty-five patients with ≥10 BMs treated with Gamma Knife stereotactic radiosurgery (GK SRS) were identified and separated into sGK vs. mGK cohorts. Survival outcomes and dosimetry data were compared between the two groups. Recursive partitioning analysis (RPA) classes were used to further stratify patients.

Results: mGK patients survived longer from the first GK treatment (p<0.009). By RPA class, patients with class 1 had a prolonged survival from BM diagnosis than those in classes 2 and 3 (p=0.004). However, survival was not significantly different between the classes from the first GK treatment (p=0.089). Stratified by mGK vs. sGK and RPA classes, sGK patients in RPA class 1 had the longest survival from BM diagnosis but the worst survival from GK treatment. mGK patients in any RPA class had the best survival from the first GK treatment. For patients with RPA class 2+3, mGK was associated with longer survival from both BM diagnosis and first treatment. Statistical but not clinical differences between the mGK vs. sGK groups were observed in the max dose to the targets and cochlea, and the V40Gy whole brain dose.

Conclusions: mGK may be beneficial if GK is initiated early at first BM diagnosis vs. sGK initiated late. Future research is required to confirm these findings and explore additional areas of interest, such as quality-of-life and economic considerations.

## Introduction

Brain metastasis (BM) is an insidious and devastating complication of systemic malignancy, afflicting around 20% of all cancer patients [1]. Although whole-brain radiation therapy (WBRT) was the standard treatment for BMs, stereotactic radiosurgery (SRS) is now generally preferred, especially in patients with limited BMs [2,3]. The literature on the efficacy of SRS in patients with multiple BMs, especially 10 or more, is limited and has often been excluded from studies [4,5]. While providing excellent local control and minimizing the neurotoxic side effects associated with WBRT, SRS may result in greater rates of intracranial relapse [6,7]. This could be addressed with salvage SRS treatments as BMs recur. However, whether utilizing multiple SRS sessions for multiple (≥10) BMs leads to similar survival outcomes compared to a single treatment is unknown. A concern with using multiple SRS sessions, especially in patients with many (≥10) BMs, is that there could be higher cumulative doses of radiation delivered to normal tissue than from a single treatment. There is currently a lack of studies that compare the survival and dosimetry differences between single and multiple SRS sessions to treat many (≥10) BMs [8].

Here, we retrospectively evaluate whether patients who received treatment for BMs, as they were discovered, through multiple sessions of Gamma Knife radiosurgery (mGK) for a total of ≥10 BMs had different survival outcomes or cumulative radiation doses when compared to those patients with ≥10 BMs treated with single-session Gamma Knife treatment (sGK).

## Materials and methods

Thirty-five patients, each with ≥10 BMs, received Gamma Knife (GK) SRS between 2008 and 2021 at a single academic center. They were separated into two groups: (1) those who underwent sGK that treated ≥10 BMs, or (2) those who underwent mGK that treated ≥10 BMs over the course of 2+ treatments. A retrospective chart review was undertaken for data related to demographic characteristics, patient survival, and dosimetry information. The center’s institutional review board approved this study (#12901) and did not require patient consent due to its retrospective nature.

Operational definitions

GK SRS was performed at our institution with the Leksell Gamma Knife (LGK) (Elekta AB, Stockholm, Sweden) model Perfexion after 2014 and before 2014 was performed using the model 4C. In both systems, patients were immobilized using a frame-based system (Model G Leksell stereotactic frame) and imaged with T1 and T2-weighted MRIs using a 1.5 T Phillips (Koninklijke Philips N.V., Amsterdam, Netherlands) scanner. Patients’ treatment plans were generated using the Leksell Gamma Plan (LGP) treatment planning system based on MR imaging and physical skull measurements. Patients who have received GK SRS are routinely followed with MRI at a two-month interval. If new lesions are identified, the treating neurosurgeon and radiation oncologist jointly determine whether a new GK SRS is reasonable. After carefully reviewing the patient’s imaging, systematic disease status, and performance status, this decision is made.

**Figure 1:**
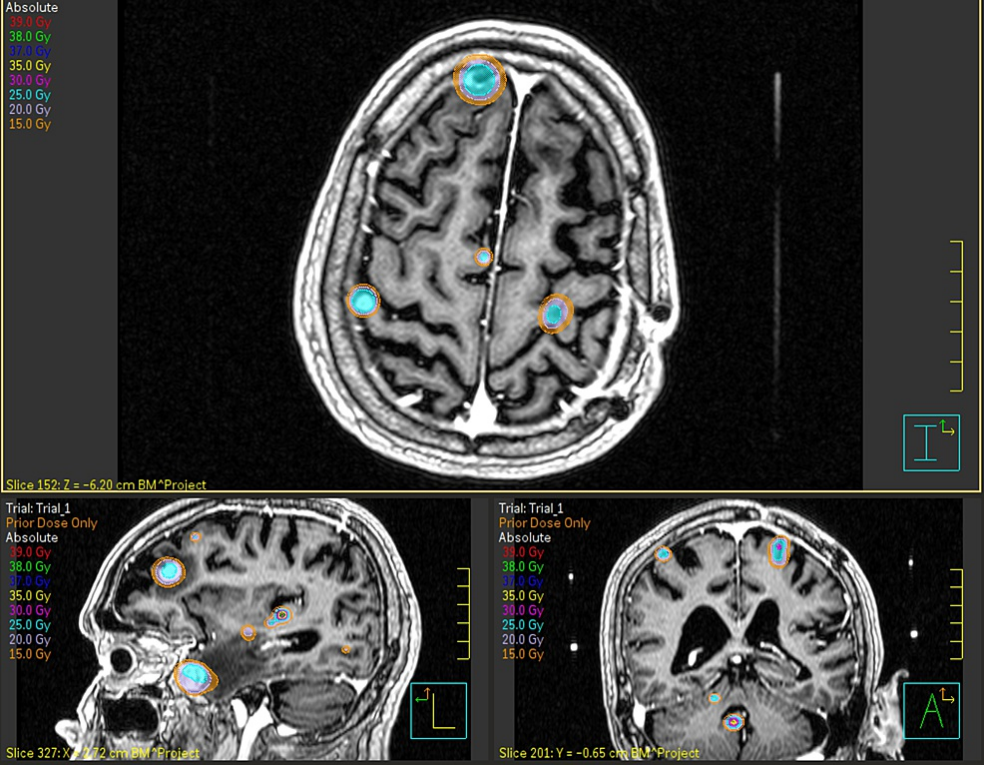
Three-view of a sample patient treated for 26 BM in three separate treatments from 2015 to 2016 BM: brain metastases

To collect radiation dosimetry data, 11 typical organs-at-risk (OAR) were contoured for each patient on T1 weighted MRI scans in LGP (version 10.1.1). All contours were approved by the neurosurgeon and/or radiation oncologist. All patient plan doses and volumes were sent to Pinnacle treatment planning system (TPS) v 16.2 (Philips Radiation Oncology Systems, Fitchburg, WI) to combine overall doses for mGK and collect total doses to the OARs. The maximum dose for all structures, the mean dose for hippocampi and whole brain, and the volume of the brain receiving 12 Gy (V12Gy) were collected. In addition, maximum, minimum, and mean prescription doses were collected. Figure 1 demonstrates a sample patient where three treatments have been collated in Pinnacle.

Demographic information retrospectively collected from medical records include age, sex, race, number of GK sessions, the total number of BMs, Karnofsky performance status (KPS) at first diagnosis, days between diagnosis of initial BM and GK treatment, and the type of primary cancer. Survival data collected include the date of first BM diagnosis, first GK treatment, and date of death. Additionally, Radiation Therapy Oncology Group (RTOG) recursive partitioning analysis (RPA)-derived prognostic classes were used to further stratify the patients by health statuses [9]. The utility of this classification system has been supported by validation studies [10]. Class 1 includes patients with a KPS >70, <65 years of age with controlled primary and no extracranial metastases. Class 3 encompasses patients with a KPS<70. Class 2 includes all other patients. The RPA class was calculated for the patient at the first BM diagnosis.

Statistical analysis

Statistical analysis was conducted using R version 4.1.1 (The R Foundation for Statistical Computing, Vienna, Austria), and tables were produced using the package gtsummary [11]. Student’s t-test or Wilcoxon rank-sum was used to compare means, and Pearson’s Chi-squared test or Fisher’s exact test was used to compare categorical variables. Kaplan-Meier curves were produced to analyze survival rates, with significance measured by the Cox proportional hazard model. Differences in radiation doses were compared with the Wilcoxon rank-sum test. Correlation studies were performed using Pearson correlation. A threshold of significance, alpha, of 0.05 was used.

## Results

A total of 35 patients received GK SRS for ≥10 BMs between 2008 and 2021; 17 received sGK, and 18 received mGK. Baseline characteristics for each group are listed in Table 1 and dates of treatment are listed in Table 2. Age at first diagnosis, sex, race, number of BMs, RPA class, KPS at first diagnosis, and primary cancer are similar between the mGK and sGK groups. The mGK group had an average of three GK sessions. The sGK cohort had a median of 12 BMs, whereas the mGK cohort had a median of 16. Patients in both groups had an average and median age of approximately 60 years, and a majority in each had an RPA class at first diagnosis of 2. Differences in mean days between BM diagnosis and GK treatment were not statistically significant, though the mGK group was treated sooner (64 days in mGK vs. 360 days in sGK). Non-small cell lung cancer accounted for most of the patient’s primary cancer diagnoses, with the second most prominent diagnosis being breast cancer.

**Table 1:** Baseline characteristics mGK: multiple Gamma Knife sessions, sGK: single session Gamma Knife, GK: Gamma Knife, RPA: recursive partitioning analysis, KPS: Karnofsky Performance Status, BM: brain metastases, NSCLC: non-small cell lung cancer, SCLC: small cell lung cancer 1: Median/Mean (SD); n (%) 2: Wilcoxon rank-sum exact test; Pearson's Chi-squared test; Fisher's exact test; Wilcoxon rank-sum test

Variable	mGK, N = 18^1^	sGK, N = 17^1^	p-value^2^
Age at first diagnosis (years)	60/62 (13)	58/58 (15)	0.52
Sex			0.40
F	11 (61%)	8 (47%)	
M	7 (39%)	9 (53%)	
Race			>0.99
Asian	2 (12%)	1 (7.7%)	
White	14 (88%)	12 (92%)	
(Missing)	2	4	
Number of GK sessions	3/3.28 (1.56)	1/1.00 (0.00)	<0.001
Total number of brain metastases	16/18 (9)	12/16 (10)	0.23
RPA class at first diagnosis			0.44
1	7 (39%)	4 (24%)	
2	10 (56%)	10 (59%)	
3	1 (5.6%)	3 (18%)	
KPS at first diagnosis	85/86 (10)	80/81 (8)	0.11
Days between diagnosis of BM and GK treatment	64/223 (226)	360/630 (902)	0.14
Primary cancer			0.75
Breast	6 (33%)	3 (18%)	
Cervical	1 (5.6%)	0 (0%)	
Choriocarcinoma	0 (0%)	1 (5.9%)	
Colon	0 (0%)	1 (5.9%)	
Melanoma	2 (11%)	4 (24%)	
NSCLC	7 (39%)	6 (35%)	
Rectal	0 (0%)	1 (5.9%)	
Renal	1 (5.6%)	0 (0%)	
SCLC	1 (5.6%)	1 (5.9%)	

**Table 2:** Treatment dates for study patients, including date range of treatment for mGK patients. sGK: single session Gamma Knife, mGK: multi-Session Gamma Knife, BM: brain metastases

	Patient #	Total no. of treated BM	No. of treatments	Treatment dates
sGK	1	10	1	2008
	2	10	1	2017
	3	11	1	2017
	4	12	1	2017
	5	11	1	2008
	6	11	1	2017
	7	15	1	2016
	8	16	1	2011
	9	17	1	2015
	10	24	1	2014
	11	27	1	2014
	12	50	1	2014
	13	11	1	2016
mGK	14	14	2	2014
	15	17	2	2016
	16	14	2	2013-2014
	18	17	3	2016-2017
	17	26	3	2015-2016
	19	25	5	2012-2015
	20	11	3	2015-2016
	21	14	3	2016-2017
	22	10	3	2011-2012
	23	10	4	2015-2016
	24	14	4	2015
	25	11	4	2014-2017
	26	10	5	2013-2018

**Figure 2:**
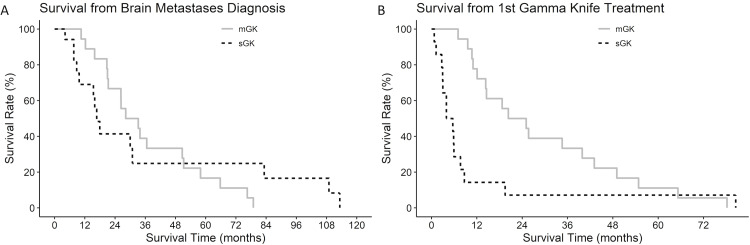
Kaplan-Meier curves of survival from (A) brain metastasis diagnosis or (B) Gamma Knife treatment; stratified by sGK vs. mGK. mGK: multiple Gamma Knife sessions, sGK: single session Gamma Knife

Kaplan-Meier survival curves were generated for each group from either the first diagnosis of BM (Figure 2A) or the first GK treatment (Figure 2B). Though differences in survival for sGK vs. mGK from BM diagnosis were not statistically significant (p=0.998, Figure 2A), mGK patients had a statistically significant longer survival from first GK treatment compared to sGK patients (p<0.009, Figure 2B).

**Figure 3:**
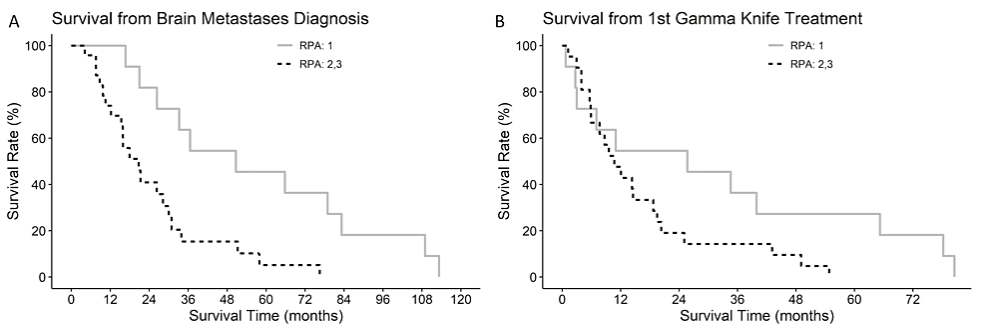
Kaplan-Meier curves of survival from (A) brain metastasis diagnosis or (B) survival from first Gamma Knife treatment, stratified by RPA class. RPA: recursive partitioning analysis

Analyzing survival by RPA class, patients with an RPA class of 1 had a longer survival from BM diagnosis than those in classes 2 and 3 (p=0.004, Figure 3A). However, survival was not significantly different between the classes from the first GK treatment (p=0.122, Figure 3B).

**Figure 4:**
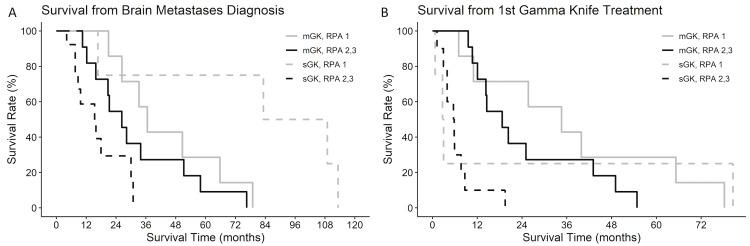
Kaplan-Meier survival curves from (A) brain metastasis diagnosis or (B) first Gamma Knife treatment are stratified by sGK vs. mGK and RPA class. mGK: multiple Gamma Knife sessions, sGK: single session Gamma Knife, RPA: recursive partitioning analysis

Survival was also stratified jointly by the number of treatments and RPA class from either the date of BM diagnosis or the date of the first GK treatment (Figures 4A, 4B). From BM diagnosis, patients who are RPA class 1 and underwent sGK had the longest survival, whereas patients who are RPA class 2+3 and underwent sGK had the worst survival. The RPA class was significant (p=0.004), while the number of treatments was not (p=0.563). Note that mGK patients in any RPA class had survival lengths between the RPA class 1 sGK and RPA class 2+3 sGK groups. From the first GK treatment, mGK patients in any RPA class had better survival rates (p=0.007), whereas sGK patients with any RPA class had the worst survival. For patients with RPA class 2+3, mGK was associated with longer survival from both BM diagnosis and first treatment compared to sGK. These results were not statistically significant when alpha=0.05 (p = 0.067).

Table 3 and Table 4 provides the differences observed between the sGK and mGK groups for dosimetry. Statistical significance between the two groups was observed in max dose to the targets, whole brain, and cochlea.

**Table 3:** Radiation dosimetry differences between the sGK and mGK groups for targets and brain. GK: Gamma Knife, WB - GTV: whole brain - gross tumor volume, mGK: multiple Gamma Knife sessions, sGK: single session Gamma Knife

			Rx dose (D95)			Whole brain dose			WB - GTV			Brainstem
# of GK Treatments		Total Tumor Volume (cc)	Min Met (Gy)	Max Met (Gy)	D95 of all mets(Gy) (weighted average)	V12 (cc)	Mean (Gy)	Max (Gy)	V12	Mean	Max	Max (Gy)
1	Mean	7.4	18.0	26.2	19.7	36.4	2.7	33.4	27.5	2.6	31.7	10.4
	Median	2.0	17.8	26.2	19.6	18.3	2.2	35.3	15.5	2.1	32.5	5.7
	Stdev	11.8	2.3	3.6	2.6	60.0	1.9	4.5	47.0	1.8	3.7	8.9
2+	Mean	7.7	18.4	35.0	21.0	41.1	3.3	48.8	33.4	3.2	42.1	13.2
	Median	6.5	17.8	30.1	20.4	32.1	2.9	41.6	24.7	2.8	37.3	8.3
	Stdev	7.0	3.1	10.8	2.4	35.1	1.7	15.9	30.3	1.6	13.6	9.7
	P -Value	0.1729	0.9313	0.0008	0.1425	0.1473	0.1841	0.0001	0.1164	0.1898	0.0013	0.2771

**Table 4:** Radiation dosimetry differences between the sGK and mGK groups for analyzed organs at risk. GK: Gamma Knife, mGK: multiple Gamma Knife sessions, sGK: single session Gamma Knife

		Optic nerve Rt	Optic nerve Lt	Chiasm	Cochlea Rt	Cochlea Lt	Lens Rt	Lens Lt	Hippo Rt		Hippo Lt	
# of GK Treatments		Max (Gy)	Max (Gy)	Max (Gy)	Max (Gy)	Max (Gy)	Max (Gy)	Max (Gy)	Mean (Gy)	Max (Gy)	Mean (Gy)	Max (Gy)
1	Mean	1.4	1.4	2.4	1.3	1.4	0.8	0.8	2.1	5.3	3.1	6.2
	Median	1.1	1.0	1.9	1.0	1.1	0.8	0.7	2.0	4.5	1.9	4.1
	Stdev	1.1	1.1	1.7	0.9	1.1	0.6	0.7	1.1	4.3	3.3	8.2
2+	Mean	2.4	2.5	3.8	2.1	2.3	1.0	1.1	3.0	7.9	3.7	8.7
	Median	1.5	1.4	2.7	1.5	1.8	0.9	0.8	2.4	4.8	2.6	5.8
	Stdev	2.6	2.3	2.6	2.2	1.6	0.5	0.6	2.5	8.7	2.9	8.2
	P -Value	0.0555	0.1290	0.0785	0.0473	0.0338	0.0555	0.0600	0.1841	0.3605	0.1784	0.184

When analyzing the correlations in the sGK and mGK groups, the dose to the OARs increases as the tumor volume increases (Tables 5, 6). The sGK group saw a negative correlation between total tumor volume and D95% prescription dose, while the mGK group did not. Also, survival from diagnosis is positively correlated to survival from the first GK treatment in the mGK group but not in the sGK group.

**Table 5:** Correlation snapshot between survival, tumor volume, and dosimetry data for a single Gamma Knife session cohort. WB - GTV: whole brain - gross tumor volume

		Survival from first Tx	Total Tumor Volume	Rx dose (D95)			Whole brain dose			WB - GTV			Brainstem	Optic nerve Rt	Optic nerve Lt	Chiasm	Cochlea Rt	Cochlea Lt	Lens Rt	Lens Lt	Hippo Rt		Hippo Lt	
				Min Met (Gy)	Max Met (Gy)	D95 of all mets(Gy) (weighted average)	V12 (cc)	Mean (Gy)	Max (Gy)	V12	Mean	Max	Max (Gy)	Max (Gy)	Max (Gy)	Max (Gy)	Max (Gy)	Max (Gy)	Max (Gy)	Max (Gy)	Mean (Gy)	Max (Gy)	Mean (Gy)	Max (Gy)
Survival from Diagnosis		-0.11	0.52	-0.40	-0.15	-0.26	0.62	0.43	-0.16	0.64	0.44	-0.22	0.29	0.42	0.52	0.43	0.38	0.30	0.45	0.53	0.26	0.00	0.23	0.06
Survival from 1st Treatment			-0.28	-0.26	-0.59	-0.28	-0.33	-0.42	-0.49	-0.33	-0.43	-0.60	-0.14	-0.25	-0.26	-0.21	-0.30	-0.23	-0.18	-0.33	-0.30	-0.25	-0.28	-0.23
Total Tumor Volume				-0.45	0.02	-0.50	0.97	0.93	-0.14	0.95	0.92	-0.04	0.29	0.89	0.72	0.79	0.63	0.62	0.83	0.74	0.79	0.20	0.46	0.21

**Table 6:** Correlation snapshot between survival, tumor volume, and dosimetry data for multiple Gamma Knife sessions. WB - GTV: whole brain - gross tumor volume

	Survival from first Tx	Total tumor volume	Rx dose (D95)			Whole brain dose			WB - GTV			Brainstem	Optic nerve Rt	Optic nerve Lt	Chiasm	Cochlea Rt	Cochlea Lt
			Min Met (Gy)	Max Met (Gy)	D95 of all mets(Gy) (weighted average)	V12 (cc)	Mean (Gy)	Max (Gy)	V12	Mean	Max	Max (Gy)	Max (Gy)	Max (Gy)	Max (Gy)	Max (Gy)	Max (Gy)
Survival from Diagnosis	0.94	0.41	0.35	0.42	0.20	0.25	0.39	0.37	0.24	0.40	0.31	-0.33	0.45	0.12	0.22	0.47	0.27
Survival from 1st Treatment		0.32	0.27	0.34	0.15	0.26	0.32	0.26	0.25	0.32	0.22	-0.47	0.31	0.09	0.07	0.29	0.15
Total Tumor Volume			0.22	0.70	0.15	0.71	0.83	0.61	0.66	0.82	0.53	-0.03	0.80	0.50	0.57	0.69	0.50

## Discussion

There is a concern with using multiple salvage SRS sessions in patients with BMs due to the potential risk of higher cumulative radiation doses. Differences in survival and radiation dosimetry between single-session and multiple-session GK SRS in patients with multiple (≥10) BMs have not been well characterized in the literature. In this study, we compared survival and dosimetry outcomes between mGK and sGK patients, finding that mGK had comparable dosimetric results to sGK.

The mGK cohort demonstrated similar survival to the sGK cohort. When comparing survival from the first GK treatment, which is impacted by how soon after BM diagnosis GK is initiated, it is not surprising that patients treated with mGK had a longer survival as they were treated with a lower disease burden. Though survival from diagnosis is not statistically different between the groups, mGK patients indicated better survival from 0 to 55 months. The mGK cohort had a shorter median number of days between diagnosis of BMs and first GK treatment (64 days) versus sGK patients (360 days) indicating other therapies were utilized prior to radiosurgery, though the retrospective nature of this study limited the ability to analyze this aspect due to lack of complete patient records. These results indicate a need for future research into investigating the potential value for early initiation of SRS for BMs with the anticipation of follow-up salvage therapies.

The use of the RPA class in our subsequent survival analyses allowed for further investigation into the effect of mGK versus sGK treatments while isolating the potential confounders of baseline patient morbidity. When separating survival data by RPA class only, the classification system works as expected, with patients in RPA class 1 having superior survival compared to those in RPA classes 2 and 3. However, there were several surprising observations when separating by RPA class and sGK versus mGK. Though sGK patients who were RPA class 1 had the best survival from the first BM diagnosis, the majority of that group had the worst survival compared to other groups when calculated from the first GK treatment. These patients had the longest delay between the first BM diagnosis and eventual GK treatment, with a median time of 1640 days (~5.5 years). Though the patients were doing well at the initial diagnosis, this might have led to using other treatment options instead of SRS. GK was most likely only initiated at end-stage disease, which did not show to be effective for survival in this cohort. Meanwhile, sGK patients in RPA class 2 or 3 presented with a more extensive disease burden (10 or more BMs), got GK treatment relatively shortly after (median of 315 days post-diagnosis), and demonstrated poor survival outcomes.

mGK patients in any RPA class indicated better survival compared to sGK patients in RPA class 2 or 3 when calculated from the first BM diagnosis and superior survival compared to sGK patients of any RPA class when calculated from the first GK treatment. These patients received GK treatment relatively early from the first diagnosis (median 64 days) and required recurrent follow-up treatments. Interestingly, the RPA class does not differentiate survival outcomes well within the mGK group. Future studies with additional patients are needed to assess factors other than RPA class to differentiate survival outcomes in mGK patients. Several hypotheses include tumor histology or neurologic deficits at baseline, though we have too few numbers when separated by RPA class and mGK to assess. It is also notable that in patients with RPA classes of 2 or 3, mGK patients trended toward significantly superior survival and a shorter median time between diagnosis and treatment (62 vs. 316 days, mGK vs. sGK, RPA 2 or 3). This trend indicates that treating patients with SRS earlier, when they have a lower BMs burden, may show a survival benefit.

There were statistical differences between the mGK and sGK groups regarding max dose (to the metastatic targets, whole brain, and cochlea). The max dose to the targets was due to either overlapping dose regions or repeated treatment to the same BM. As a result, the whole brain max dose exceeded 40 Gy in the mGK group, which has been associated with an increased probability of developing radiation necrosis [12,13]. Due to the steep dose gradient observed in GK treatments, the differences in peripheral OARs are due to BMs location. As the number of targets and treatments increases, the likelihood of targeting a region of the brain near these critical structures increases, yet we demonstrate that the dose to these structures remains well below published constraints, even with many lesions [14]. In the correlation data, the dose to the critical structures increases as the tumor volume increases. The sGK group saw a negative correlation between total tumor volume and D95% prescription dose, while the mGK group did not, likely due to treatment length becoming a concern in the sGK group. Due to the decay of Cobalt-60, BM treatments of ≥10 BMs can exceed 3 hours, especially with older cobalt sources [15]. Therefore, the prescribed dose is often decreased to reduce treatment length. Furthermore, survival from BM diagnosis is positively correlated to survival from the first GK treatment in the mGK group but not in the sGK group. This is consistent with the fact that the mGK group had their first GK treatment 64 days after diagnosis of BM, while the sGK group had GK treatment a median of 360 days after diagnosis of BMs. Therefore, GK was not part of the care plan until much later in these patients’ disease. GK is attractive at this point of care as it is a single day of treatment as opposed to other options, which require the patient to come in for many days to weeks.

There are several limitations to this retrospective study, including missing data and a lack of randomization between groups. As a result of non-randomization, it is challenging to differentiate survival benefits from selection bias. Furthermore, statistical analyses are restricted by the small number of patients, with 35 total patients, 18 of whom received mGK and 17 sGK. Patients with greater than or equal to 10 BMs treated with SRS are rare, even at a large academic institution that retrospectively reviewed all such patients over 13 years (2008-2021). This dataset presents an essential contribution to the literature where data needs to be improved. Future work should involve gathering more survival data on patients at other academic institutions that perform GK SRS in patients with multiple BMs. Additionally, the financial and quality-of-life issues associated with mGK are not considered. We also did not explore neurocognitive aspects related to treatment. Future studies should aim to address these topics.

## Conclusions

Our results demonstrate that there may be a potential survival benefit for cancer patients with BMs if GK treatment is initiated early at first BM diagnosis. The slight differences in the dose delivered to most of the critical structures indicate it is reasonable to treat such patients over multiple sessions or treat BMs upon first discovery with the option of salvage treatments as required. However, more work is needed to examine the impact of multiple treatments on the volume of the brain receiving 40 Gy or more. Future research should also confirm these findings with larger patient populations and explore additional areas of interest, such as quality of life and economic burdens.
